# Study protocol to test the efficacy of self-administration of dexmedetomidine sedative therapy on anxiety, delirium, and ventilator days in critically ill mechanically ventilated patients: an open-label randomized clinical trial

**DOI:** 10.1186/s13063-022-06391-w

**Published:** 2022-05-16

**Authors:** Linda L. Chlan, Craig R. Weinert, Mary Fran Tracy, Debra J. Skaar, Ognjen Gajic, Jessica Ask, Jay Mandrekar

**Affiliations:** 1 Mayo Clinic, Rochester, MN USA; 2 University of Minnesota, Minneapolis, MN USA

**Keywords:** Mechanical ventilation, Sedation, Anxiety, Clinical trial, Intensive care

## Abstract

**Background:**

Administration of sedative and opioid medications to patients receiving mechanical ventilatory support in the intensive care unit is a common clinical practice.

**Methods:**

A two-site randomized open-label clinical trial will test the efficacy of self-management of sedative therapy with dexmedetomidine compared to usual care on anxiety, delirium, and duration of ventilatory support after randomization. Secondary objectives are to compare self-management of sedative therapy to usual care on level of alertness, total aggregate sedative and opioid medication exposure, and ventilator-free days up to day 28 after study enrolment. Exploratory objectives of the study are to compare self-management of sedative therapy to usual care on 3- and 6-month post-discharge physical and functional status, psychological well-being (depression, symptoms of post-traumatic stress disorder), health-related quality of life, and recollections of ICU care. ICU patients (*n* = 190) who are alert enough to follow commands to self-manage sedative therapy are randomly assigned to self-management of sedative therapy or usual care. Patients remain in the ICU sedative medication study phase for up to 7 days as long as mechanically ventilated.

**Discussion:**

The care of critically ill mechanically ventilated patients can change significantly over the course of a 5-year clinical trial. Changes in sedation and pain interventions, oxygenation approaches, and standards related to extubation have substantially impacted consistency in the number of eligible patients over time. In addition, the COVID-19 pandemic resulted in mandated extended pauses in trial enrolment as well as alterations in recruitment methods out of concern for study personnel safety and availability of protective equipment. Patient triaging among healthcare institutions due to COVID-19 cases also has resulted in inconsistent access to the eligible study population. This has made it even more imperative for the study team to be flexible and innovative to identify and enrol all eligible participants. Patient-controlled sedation is a novel approach to the management of patient symptoms that may be able to alleviate mechanical ventilation-induced distress without serious side effects. Findings from this study will provide insight into the efficacy of this approach on short- and long-term outcomes in a subset of mechanically ventilated patients.

**Trial registration:**

ClinicalTrials.gov NCT02819141. Registered on June 29, 2016.

## Administrative information

Note: The order of the items has been modified to group similar items (see http://www.equator-network.org/reporting-guidelines/spirit-2013-statement-defining-standard-protocol-items-for-clinical-trials/).

**Table:** 

Title {1}	Study protocol to test the efficacy of self-administration of dexmedetomidine sedative therapy on anxiety, delirium, and ventilator days in critically ill mechanically ventilated patients: an open-label randomized clinical trial
Trial registration {2a and 2b}	2a. ClinicalTrials.gov NCT02819141 Registered 6/29/2016 IND#111693 (C. Weinert) 2b. the ClinicalTrials.gov registry meets the WHO International Clinical Trials Registry Platform criteria https://clinicaltrials.gov/ct2/manage-recs/resources#InternationalCommittee
Protocol version {3}	12/13/2021 version 22
Funding {4}	The project described is supported by funding from the National Heart, Lung, and Blood Institute, National Institutes of Health (5R01 HL130881) and by the Clinical and Translational Science Award (CTSA) programme, through the National Institutes of Health (NIH) National Center for Advancing Translational Sciences (NCATS), grant UL1TR002373. The content is solely the responsibility of the authors and does not necessarily represent the official views of the NIH.
Author details {5a}	Chlan, Linda L. Mayo Clinic, Rochester, MN U.S.A. Weinert, Craig R. University of Minnesota, Minneapolis, MN Tracy, Mary Fran University of Minnesota, Minneapolis, MN Skaar, Debra J. University of Minnesota, Minneapolis, MN Gajic, Ognjen Mayo Clinic, Rochester, MN Ask, Jessica Mayo Clinic, Rochester, MN Mandrekar, Jay Mayo Clinic, Rochester, MN
Name and contact information for the trial sponsor {5b}	This research is supported by the National Heart, Lung, and Blood Institute of the National Institutes of Health (telephone 1-877-645-2248, award No. 5R01 HL130881).
Role of sponsor {5c}	The funders of this study had no role in the study design; collection, management, analysis, and interpretation of the data; writing of this report; or the decision to submit the report for publication, and they have no authority over any of these activities.

## Introduction

### Background and rationale {6a}

Administration of sedative therapy to critically ill patients receiving mechanical ventilatory support in the intensive care unit (ICU) is common practice. Two of the most common indications for the administration of sedative medications are to reduce anxiety and promote tolerance of mechanical ventilation [1–3]. While indicated at times to promote patient synchrony with mechanical breaths, these potent medications are not without serious short- and long-term side effects [2]. Common short-term side effects of sedative medications include respiratory drive suppression and reduced level of consciousness. More long-term side effects of these medications include prolonged duration of mechanical ventilation due to delayed spontaneous breathing trials, immobility, and prolonged stay in the ICU. Because of these side effects, sedative therapy has evolved over the past decade from heavy sedation so patients can “sleep” through their critical illness or injury without recall to administration of opioids for an analgo-sedation approach and the absence of sedative medication administration during ventilatory support. The common denominator in these varying approaches to sedative therapy is the reliance on clinicians to manage the administration of these medications. Nurses are responsible for the management of patients and their symptoms, as well as the administration of medications to facilitate synchrony with the ventilator. However, sedative medications are typically not administered based on individual symptoms but based on the patient’s alertness as commonly assessed in the ICU by the Richmond Agitation-Sedation Scale (RASS) [2, 4]. Novel approaches to the management of patient symptoms that alleviate mechanical ventilation-induced distress without serious side effects are needed. Thus, the purpose of this study is to test the efficacy of patient self-administered sedative therapy on select short- and long-term outcomes.

### Objectives {7}

The primary objective of the study is to assess the efficacy of patients’ self-management of sedative therapy using dexmedetomidine compared to usual sedation practices in mechanically ventilated subjects. Efficacy of self-management of sedative therapy is defined by statistically significant differences compared to usual sedation care for (a) anxiety level over time after randomization, (b) incidence of delirium after randomization, and (c) duration of mechanical ventilation after randomization. Secondary objectives of the study are comparing self-management of sedative therapy to usual sedation care on the following: (a) level of alertness over time after randomization, (b) total aggregate sedative and opioid medication exposure over time, and (c) ventilator-free days up to day 28 after study enrolment. Exploratory objectives of the study comparing self-management of sedative therapy to usual sedation care on 3- and 6-month post-ICU outcomes include physical and functional status, psychological well-being (depression, symptoms of post-traumatic stress disorder), health-related quality of life, and recollections of ICU care.

### Trial design {8}

A two-group, open-label randomized multi-centre clinical trial superiority design will address the study objectives to test the efficacy of self-management of sedative therapy on primary, secondary, and exploratory outcomes. Patients are randomly allocated in a 1:1 ratio to either (1) experimental group of self-management of sedative therapy using dexmedetomidine or (2) standard of care control group of nurse-administered sedative therapy based on blocks stratified by site developed by the study biostatistician.

## Methods: participants, interventions, and outcomes

### Study setting {9}

Critically ill patients receiving mechanical ventilatory support are recruited from two academic medical centres and two community hospitals in the Midwestern State of Minnesota (MN), USA. The first site is St Marys Hospital on the Mayo Clinic Rochester, MN, campus with 80 adult ICU beds consisting of medical (32 beds), surgical (18 beds), trauma (12 beds), and neurological (18 beds) ICUs. The other participating sites are as follows: the M Health Fairview, University of Minnesota Medical Centre (UMMC) Medical, Surgical/Neurological and Cardiovascular ICUs in Minneapolis, MN. UMMC is an academic medical centre and the primary teaching hospital of the University of Minnesota Medical School. Combined, these adjacent ICUs have 62 staffed beds. The first participating community hospital is M Health Fairview Southdale Hospital, Edina, MN, which has a mixed medical-surgical/neurological/cardiovascular surgery ICU with 22 beds. M Health Fairview Ridges Hospital, Burnsville, MN, has a medical-surgical ICU with 12 beds. The two community hospitals have physician intensivist providers from the same ICU programme as UMMC thereby ensuring that the general ICU practice regarding sedation and mechanical ventilation management is like that of UMMC.

### Eligibility criteria {10}

#### Pre-screening

The electronic health record (EHR) is screened daily to identify patients receiving mechanical ventilatory support who are eligible for further evaluation in one of the participating ICUs. The EHR automatically places an electronic flag designating any ICU patients with a laboratory-confirmed COVID-19-positive test. Any of these patients receiving mechanical ventilation are not eligible for this trial until they are considered non-contagious per hospital infection control regulations, generally 3 weeks after symptom onset.

Potential subjects are first evaluated by the trained research staff for participation with a bedside pre-screening test to evaluate motor abilities, alertness, and for the presence of acute confusion (delirium). Motor ability is assessed by placing the medication push-button activation device button in the patient’s hand and asking them to depress the button or asking a patient to click a ball-point pen with his/her thumb. This motor test is used to verify the hand strength needed to depress the medication self-delivery button. The research staff then complete an alertness screen which assesses the patient’s ability to communicate and appropriately follow verbal commands accurately. The two-step process of the Confusion Assessment Method-ICU (CAM-ICU) [5] is administered to determine a patient’s alertness and for the presence of acute confusion (delirium). Step 1 consists of arousal and alertness assessment using the RASS [4]. Step 2 includes administration of the CAM-ICU which consists of the delirium assessment component. The dichotomous result is either delirium absent (CAM-ICU negative) or delirium present (CAM-ICU positive). Patients are required to be assessed as RASS level of − 2 to + 1 to be eligible for study participation.

##### Inclusion criteria

Patients may be included in the study if they meet all of the following inclusion criteria: (a) acutely mechanically ventilated during the index hospitalization; (b) currently receiving a continuous intravenous infusion of a sedative/opioid medication(s) or have received at least one intravenous bolus dose of a sedative/opioid medication in the previous 24 h (fentanyl, hydromorphone, ketamine, morphine, midazolam, diazepam, lorazepam, propofol, haloperidol, dexmedetomidine); (c) pass the pre-screening test and are assessed RASS − 2 to + 1; and (d) age ≥ 18 years old.

##### Exclusion criteria

Critically ill mechanically ventilated patients are excluded from the study if any of the following conditions exist: (a) aggressive ventilatory support or prone positioning; (b) hypotension (systolic blood pressure < 85 mmHg) requiring a vasopressor medication at a dose of norepinephrine or epinephrine > 0.15 mcg/kg/min or vasopressin > 2.4 units per hour. Patients are excluded if they require more than one continuous infusion of a catecholamine vasopressor medication simultaneously and are excluded if the vasopressor dose was higher than norepinephrine or epinephrine 0.15 mcg/kg/min, vasopressin > 2.4 units per hour, phenylephrine > 3 mcg/kg/min, dopamine > 10 mcg/kg/min, or dobutamine at any dose in the prior 6 h. In addition, patients are excluded if dopamine is being used to increase heart rate; (c) second- or third-degree heart block or bradycardia (heart rate < 50 beats/min); (d) paralysis or other condition preventing the use of push-button device; (e) positive pregnancy test or lactation; (f) acute hepatitis or acute liver failure, (g) acute stroke or uncontrolled seizures; (h) acute myocardial infarction within 48 h prior to enrolment; (i) severe cognition or communication problems (such as coma, deafness without signing literacy, physician-documented dementia); (j) chronic mechanical ventilator support in place of residence prior to current hospitalization; or (k) imminent extubation from mechanical ventilator support in the opinion of the clinical ICU team.

### Who will take informed consent? {26a}

The trained research staff enrol those eligible patients who pass the pre-screening test. Signed informed consent is obtained from either the patient or via proxy consent of the patient’s legally authorized representative. If a patient passes the pre-screening test, RASS − 2 to + 1 except is found to be CAM-ICU positive (delirium present), proxy consent must be obtained. In the case of proxy consent, patients must be willing and able to self-medicate and provide verbal assent with an affirmative head nod “yes”.

### Additional consent provisions for collection and use of participant data and biological specimens {26b}

Access to and permission to collect study-relevant personal health information from the EHR are obtained from all subjects. The Mayo Clinic site includes this permission in the informed consent document, whereas the M Health Fairview sites utilize a separate consent document for personal health information.

### Interventions

#### Explanation for the choice of comparators {6b}

We selected dexmedetomidine as the sedative for patient self-administered sedation due to its pharmacologic and pharmacokinetic properties. Dexmedetomidine is a selective alpha-2 adrenergic agonist with more lightly sedating properties than another commonly used ICU sedative, propofol. Patients receiving dexmedetomidine can be easily awakened and, thus, are more likely to be capable of meeting their sedative needs with a self-controlled device. Benzodiazepines, such as midazolam, have active metabolites that can accumulate in kidney impairment and result in unpredictably prolonged sedation [1]. Dexmedetomidine has a rapid distribution half-life of 6 min and terminal elimination half-life of on average 2 h and linear pharmacokinetics within the usual dosage range of 0.2 to 0.7 mcg/kg/h [6]. These characteristics ensure a rapid clinical effect in response to patients’ bolus self-administration for anxiety and facilitate nursing adjustment of the basal infusion based on the number of boluses administered the preceding 2 h. This study is performed under the approval of the United States Food and Drug Administration (FDA) Investigational New Drug number 111693 (C.R.W.) since dexmedetomidine is only FDA-approved for 24 h by continuous infusion. Preparation and distribution of the drug are the responsibility of the Mayo Clinic and UMMC Investigational Drug Service pharmacies.

#### Intervention description {11a}

##### Experimental condition self-management of sedative therapy

We use a continuous basal infusion (0.2–0.7 mcg/kg/h) with 3 allowable patient-controlled self-boluses per hour (0.25 mcg/kg) each with a 20-min lock-out [7]. Standard infusion pumps with a push-button device already in clinical practice are used to administer the dexmedetomidine protocol. The Lifecare PCA® Infusion System was used at both sites initially until the M Health Fairview sites switched to the CADD Solis® infusion pump system in 2019. Set-up and management of the infusion pumps and titration of basal infusion are the sole responsibility of the patient care nursing staff, though study coordinators are present to answer questions and verify protocol orders. The infusion pump is set in the PCA + continuous mode. Intermittent patient self-initiated dexmedetomidine doses are delivered in 1 mL over 35 s. Subjects in the experimental group are prompted to use the push-button device when feeling anxious. Delivery accuracy is ± 5% for continuous delivery rates > 1 mL/h. Settings, dose delivery times, and aggregate dosing are recorded by the pump for later retrieval. The basal infusion is adjusted by the patient care nurse per protocol every 2 h based on the number of sedative self-administered administered in the previous 2 h. Details on the dexmedetomidine basal infusion, titration algorithm, and dosing according to a subject’s most recent daily weight on enrolment are described elsewhere [7].

##### Standard of care control condition nurse-administered sedative therapy

Subjects randomized to the control condition receive standard care for the respective ICU which consists of nurse-administered sedative therapy as ordered by the primary care team.

#### Criteria for discontinuing or modifying allocated interventions {11b}

Daily adverse event monitoring is performed for subjects randomized to either the treatment or control condition. A trained member of the research team reviews the EHR for the presence of hypotension, bradycardia, delirium, self-extubation, and protocol deviations related to drug, pump, or both. Heart rate and blood pressure are abstracted from the EHR. The research staff or ICU nurses caring for subjects alert a member of the research team and notify the study physician for sustained (lasting > 30 min) systolic blood pressure < 80 or > 180 mmHg, diastolic < 50 or > 100 mmHg, heart rate < 40 or > 120 beats/min,; persistent inability to understand the rationale for triggering the push-button PCA device despite education and demonstration, or marked worsening of respiratory status requiring aggressive ventilatory support with deep sedation and/or chemical paralysis. The subject’s condition is reviewed by a designated study physician with the primary care team for any necessary intervention including pausing the research protocol or withdrawal of the subject from the medication part of the protocol. Even if the medication part of the self-management of the sedative therapy arm is stopped, we continue to collect three-times daily assessments (7 am, 1 pm, and 7 pm ± 2 h), sedative drug exposure and post-intubation subject questionnaires to permit “intention-to-treat” analyses.

#### Strategies to improve adherence to interventions {11c}

Three main strategies are used to promote adherence to the protocol by the ICU nursing staff. First, the protocol-directed three-times daily assessments of all subjects provide regular check-in with the responsible patient care nurse. The experimental protocol is reviewed, the dexmedetomidine titration and patient management guidelines are reinforced, and the nurse is queried about any questions or concerns. Secondly, a 3-ring binder is maintained at the subject’s bedside containing protocol reference materials such as brief information on the study specifics including hypotension alert parameters, contact numbers for research personnel, and directions for completing the supplemental medication log. Lastly, our formal intervention fidelity monitoring plan utilizes checklists to track adherence to the experimental and control group conditions.

#### Relevant concomitant care permitted or prohibited during the trial {11d}

Supplemental sedative (midazolam, haloperidol) and analgesic medications (fentanyl, morphine sulphate) are allowed for experimental group subjects; choice and dosage are standardized between sites and ordered at the discretion of the study physician writing the medication orders based on the subject’s current medical condition, current medications, and plan of care. Nurses can administer these additional sedative/analgesic medications as deemed necessary to address patient needs. Any supplemental medications and the reason(s) for administration are documented on a paper study tracking log at the bedside in addition to EHR standard documentation. There are no care recommendations or protocols instituted for subjects randomized to the usual care control condition.

#### Provisions for post-trial care {30}

Subjects do not receive any monetary remuneration for study participation. As detailed in our informed consent documents, any injury or harm resulting from participating in this clinical trial is billed to the patient’s insurance as with usual care.

### Outcomes {12}

There are two phases of this clinical trial, the ICU phase up to 7 days and the 6-month post-ICU recovery phase. The primary and secondary outcomes are focused on the ICU phase of the clinical trial with the analysis metric of the mean/median changes over time from baseline study entry randomization through ICU day 7 or until extubation, transfer/discharge from the ICU, withdrawal of consent, removal from the study, or death.

The three individual, primary outcomes are as follows. Patient-reported anxiety intensity level over time is measured three times daily with a visual analogue scale-anxiety (VAS-A). The VAS-A is a valid, non-burdensome single-item instrument to measure anxiety intensity over repeated assessments in mechanically ventilated patients [8–11]. VAS-A intensity scores range from 0 to 100 mm. The VAS-A is presented to patients with a vertical orientation, similar to a thermometer, with anchors on each end of designating *not anxious at all* = 0 to *most anxious ever* = 100. Patients mark their current level of anxiety intensity on the VAS-A line, resulting in a score from 0 to 100. The duration of mechanical ventilation is measured in the mean/median number of days after randomization to extubation. Lastly, the presence/absence of delirium after randomization is assessed three times daily by the CAM-ICU tool.

The rationale for focusing the primary outcomes on patient-reported anxiety, duration of mechanical ventilation, and delirium is that the study intervention (patient-controlled sedation with dexmedetomidine) should not have long-lasting effects after dexmedetomidine is stopped after extubation. The elimination half-life of dexmedetomidine is on average 2 h. The hypothesized mechanism whereby self-management of sedation therapy reduces mechanical ventilation duration is that, compared to clinician-administered sedation, the subject is more awake, less delirious and less anxious, and therefore more likely to undergo and/or pass a weaning trial (pressure support trial or similar test of spontaneous breathing with adequate mental status) in the clinicians’ judgement. There is no evidence to suggest that self-management of sedative therapy with dexmedetomidine would affect the underlying course of the disease that led to intubation or to have long-lasting effects on chronic conditions that may prevent further intubations weeks after the intervention is completed.

The first secondary outcome is change in the level of alertness of subjects by the group after randomization and for up to 7 days during the ICU stay. Alertness is assessed three times daily using the RASS, yielding ordinal/rank data from RASS − 5 to + 4. Sedative exposure is measured daily for up to 7 days during the ICU stay and will be analysed for mean/median change over time. Sedative exposure is operationalized as a daily aggregate measure of sedation frequency and sedation intensity based on receipt of 9 commonly administered intravenous sedative and analgesic medications (lorazepam, midazolam, propofol, morphine, hydromorphone, fentanyl, dexmedetomidine, haloperidol, ketamine) for up to 7 days after enrolment. This method allows aggregation across a study sample of weight-adjusted doses of sedatives and analgesics and anti-psychotics for which there are no pharmacologically valid conversion “equivalents”. Details on calculating sedative exposure are found elsewhere [12].

Lastly, ventilator-free days (VFD) are defined as the mean/median number of days between successful weaning from mechanical ventilation and day 28 after randomization. We count VFD only during the current hospitalization. For patients receiving a tracheostomy to assist in weaning, “extubation” is defined as the first hour of a consecutive 24 h or more interval when the patient is on no positive pressure ventilator support. Patients can still be connected to the ventilator tubing to receive humidified gas or oxygen supplementation but not receiving PEEP or pressure support. Patients alive and on the ventilator at the time of hospital discharge (all of these would be ventilated via tracheostomy) are considered to be alive and on the ventilator for the remainder of the 28 days after randomization. Patients alive and off the ventilator at the time of hospital discharge are considered to be alive and off the ventilator for the remainder of the 28 days after randomization. Since one of the study sites (Mayo Clinic) has an internal chronic respiratory support unit, a patient transferred to that unit does not count as a hospital discharge and ventilator days are counted up to day 28. Some patients are “terminally extubated” but do not die immediately. In that case, we do not count the hours or days alive after terminal extubation as an “alive and vent-free day.” Some subjects have multiple episodes of intubation within the incident hospitalization in which case we aggregate all of those ventilator days in the VFD calculation. We do not include non-ICU times of ventilation such as operating room or PACU. If an enrolled subject comes to the ICU from the OR intubated, then those ICU ventilator hours or days count towards VFD whether or not the procedure was emergency or elective as long as it occurred within the initial hospitalization. If the subject is discharged from the hospital and returns for more ventilator support, those days are not counted in the VFD calculation.

The first exploratory outcome is post-extubation recall obtained prior to hospital discharge and at 3 and 6 months with the 25-item Intensive Care Experience (ICE) Questionnaire. The ICE Questionnaire contains four main categories: awareness of surroundings, frightening experiences, recall of experiences, and satisfaction with care [13]. ICE mean/median scores obtained at each of the three assessment time points will be compared by group for change over time. Subjects who are not extubated prior to ICU transfer or hospital discharge are not assessed for ICU recall.

The following post-ICU outcomes are obtained via telephone at 3 and 6 months after the date of study randomization and are analysed for mean/median change over time by group. (1) Physical and functional status is assessed by the 6-item Katz Activities of Daily Living Scale (KADL) [14]; (2) the 10-item Functional Activities Questionnaire (FAQ) assesses instrumental activities of daily living that require higher-order abilities, such as cooking, driving, managing finances, and medications; and (3) psychological well-being is assessed with two instruments: (a) the 9-item Patient Health Questionnaire (PHQ-9) a brief, 9-item tool that measures cardinal symptoms of depression [15], and (b) symptoms of post-traumatic assessment disorder are assessed with the Posttraumatic Stress Disorder Checklist Event Specific (PCL) [16]. Lastly, health-related quality of life is assessed with the Short Form-36 (SF-36) [17].

#### Participant timeline {13}

**Fig. 1:**
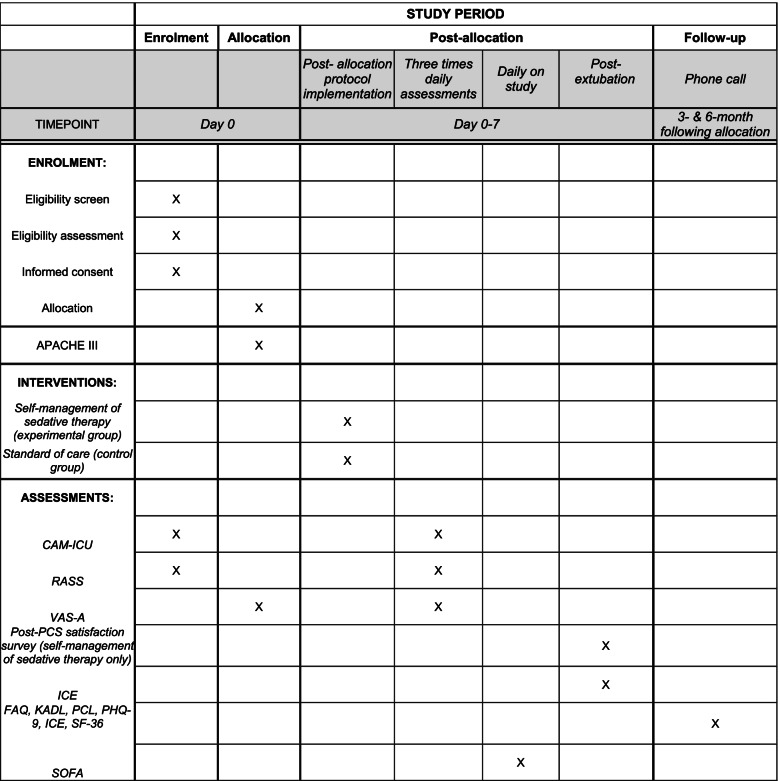
Schedule of enrolment, interventions, and assessments. CAM-ICU, Confusion Assessment Method-ICU; RASS, Richmond Agitation- Sedation Scale; VAS-A, Visual Analog Scale-Anxiety. KADL, Katz Activities of Daily Living Scale; PHQ-9, Patient Health Questionnaire; PCL, Posttraumatic Stress Disorder Checklist Event Specific; The Short Form-36 (SF-36, ); FAQ, Functional Activities Questionnaire; ICE, Intensive Care Experience Questionnaire. SOFA, Sequential Organ Failure Assessment; Acute Physiology; APACHE, Age and Chronic Health Evaluation

The participant timeline is presented in Fig. 1.

#### Sample size {14}

Our primary outcomes are anxiety, duration of mechanical ventilation, and presence of delirium. Each primary outcome is analysed separately. In our preliminary study [18], anxiety decreased over time for the experimental self-management of sedative therapy group by 5 points from 58.1 to 53.1 (0–100 mm visual analogue scale), whereas it increased by 15.5 points from 43.7 to 53.9 for the control group. The calculated delta change for anxiety = .45 for an effect size = .11 with a sample size of 95 per group (190 total). We base our target sample size on these anxiety data for this efficacy RCT. During the pilot RCT, no patients randomized to the experimental group became delirious, while four patients randomized to the control group developed delirium (*p =* .058). A sample of 35 subjects per group would be required (70 total). The power calculation for the duration of mechanical ventilatory support after study enrolment (Mann-Whitney *U*) with an effect size of .41 would = 43 per group (86 total). We estimated the power for multilevel models approximating our study design. A sample size of 95 patients per arm, 190 total (at a minimum of 7 data collection points for each, resulting in ~ 1050 observations) will have greater than 80% power to detect small to moderate effects (i.e. 0.11 or greater) in between-group differences in anxiety, delirium, and duration of mechanical ventilation at alpha = .05 for all proposed models.

#### Recruitment {15}

Registered nurse study coordinators are present in the participating ICUs daily to screen potentially eligible patients and to interact regularly with the staff. This is an ICU-based clinical trial whereby enrolment is contingent upon patient census and whether patients meet our strict inclusion criteria to conduct this efficacy trial safely and appropriately. Study coordinators are trained by the investigators to approach patients calmly and in an unhurried manner; use short, simple sentences that contain one idea at a time; and provide patients with a means of communication via paper/pen for note writing or a letter board. A brochure that provides an overview of the clinical trial is provided to patients and their family visitors that serve as a non-threatening study introduction prior to seeking informed consent.

### Assignment of interventions: allocation

#### Sequence generation {16a}

Randomization lists for each site were generated by the study statistician using computer-generated random numbers. A stratified block randomization approach with a block size of 2 was used for this open-label study. Each participating research site is the stratification factor.

#### Concealment mechanism {16b}

Randomization lists for each participating study site are incorporated into the Research Electronic Data Capture (REDCap®) database, accessible only to authorized study coordinators at each site after study eligibility is confirmed. Given this is an open-label clinical trial, for patients randomized to the experimental arm, each site’s central pharmacy prepares and dispenses the dexmedetomidine bar-coded medication cartridges.

#### Implementation {16c}

Authorized study coordinators enrol and assign participants to the experimental intervention arm or the control arm based on the allocation sequence included in the study’s REDCap® database.

### Assignment of interventions: blinding

#### Who will be blinded {17a}

This is an open-label efficacy clinical trial without blinding of ICU staff to either the experimental or control conditions. Research personnel responsible for screening, enrollment and data collection are not blinded.

#### Procedure for unblinding if needed {17b}

Given this is an open-label efficacy clinical trial, procedures for unblinding are not applicable.

### Data collection and management

#### Plans for assessment and collection of outcomes {18a}

##### Study entry demographic and clinical data

Data are abstracted from the EHR or from the subject on the following: age, sex, race, ethnicity, ICU admission and day of enrolment weight, medical diagnoses, indication for ventilatory support, medications including sedative medications in the prior 24 h, ventilator settings, and severity of illness measured by Acute Physiology, Age and Chronic Health Evaluation III (APACHE III) score [19]. The APACHE III is used to determine the severity of illness during the first 24 h of ICU admission. For daily illness severity, a Sequential Organ Failure Assessment (SOFA) [20] is scored each day from the medical record for the length of study enrolment. Any differences in illness severity, SOFA scores, age, or biological sex will be considered as covariates in subsequent analyses. Data abstraction, data entry, and assessments are completed by study coordinators or trained designees. Case report forms (CRFs) are kept in a binder at the bedside for each study participant where primary outcomes for patient-reported anxiety (VAS-A), the incidence of delirium (CAM-ICU), and secondary outcome, level of alertness over time (RASS), are assessed three times daily while in the active ICU study phase (see Fig. 1 for the schedule of events, interventions, and assessments). CRFs are entered into REDCap® by trained study staff.

##### Assessment and collection of outcomes

The primary outcomes are as follows:

- Patient-reported anxiety level over time after randomization
- Incidence of delirium after randomization
- Duration of mechanical ventilation after randomization

The secondary outcomes are as follows:

- Level of alertness over time after randomization
- Total aggregate sedative and opioid medication exposure over time
- Ventilator-free days after weaning from mechanical ventilation and day 28 after study enrolment

The exploratory outcomes are as follows:

- Physical and functional status
- Psychological well-being (depression, symptoms of post-traumatic stress disorder)
- Health-related quality of life
- Recollections of ICU care and experiences

#### Plans to promote participant retention and complete follow-up {18b}

During the ICU phase of this clinical trial, per-protocol patients are assessed in-person three times daily by trained research staff for anxiety, level of alertness, and the presence/absence of delirium. The regular presence of a member of the research team on the ICUs greatly contributes to participant retention while addressing any issues or concerns in real-time. The 3- and 6-month post-ICU assessments for the exploratory aims are completed by telephone. A contact telephone number is recorded prior to hospital discharge. A reminder call is placed to patients by a member of the research team in advance of the 3- and 6-month assessment; questionnaires may be sent in the postal mail or electronically to facilitate completion over the telephone. Adequate time is provided during telephone assessment calls to accommodate a participant’s energy level.

#### Data management {19}

Research Electronic Data Capture (REDCap®) is the electronic database used for all data entry. Access is by individual permission only, restricted to members of the research team, and is password protected through individual email accounts. Study coordinators or trained designees abstract patient data from the EHR and enter CRFs into REDCap®. Study coordinators and staff are all onsite, trained individually, and recorded on a delegation of authority log retained in a regulatory binder and signed by the principal investigator at each site. The study is monitored biannually for compliance and quality assurance. The monitor has access to the EHR, CRFs, regulatory documents, and REDCap® for use in verifying information. All entered data on the primary study outcomes and sedative exposure are audited independently from the individual who entered the original data. Case report forms (CRFs) are stored in a locked file cabinet separate from signed informed consent forms. Both study sites are transitioning to electronic CRFs in the next year. REDCap® has built-in alerts for value and range entries to notify staff of out-of-range values. Edits made to data entry are recorded in the system history as a change and are tracked throughout the study. User privileges and access in REDCap® are monitored and granted by the database manager. User rights are updated with study staff changes. The manual of operations is maintained to provide a consistent data management plan and improve accuracy.

#### Confidentiality {27}

A screening filter is used when accessing the EHR for screening to identify only those ICU patients receiving mechanical ventilatory support. The only study documents that contain a patient’s identifying information are the signed consent form and the permission to obtain protected health information document. These documents are stored in a locked file cabinet and stored for seven years. After that time, all study documents are shredded.

#### Plans for collection, laboratory evaluation, and storage of biological specimens for genetic or molecular analysis in this trial/future use {33}

There are no biological specimens collected for this clinical trial.

## Statistical methods

### Statistical methods for primary and secondary outcomes {20a}

#### Primary aims

The primary aims of the study are to determine the efficacy of self-management of sedative therapy in ICU patients receiving mechanical ventilation compared to nurse-administered sedative therapy on (1) anxiety, (2) presence of delirium, and (3) duration of mechanical ventilation. All three outcomes will be assessed individually with multilevel models. Multilevel growth curve analyses controlling for illness severity, age, sex, and sedative exposure will be used to model the trajectories of anxiety as predicted by group among all patients. The level 1 sub-model will estimate how each patient’s anxiety changes over the 7-day ICU study period. The level 2 sub-model will relate the inter-individual differences to the intervention group and other time-invariant predictors (such as biological sex) and will estimate a subject’s initial anxiety level and rate of change in anxiety over the study period. To account for correlation within patients over time, we will model anxiety with the subject as a random effect. Effect of time will be included as a linear or non-linear term based on the model fit. Subsequent models will contain time-varying covariates [i.e., sedative exposure and illness acuity (APACHE III)]. This will allow us to focus on within-subject effects, that is, whether within-subject differences in the covariates are associated with within-subject differences in anxiety, delirium, and duration of mechanical ventilation. A choice of an appropriate covariance structure for the residuals will be made based on a model fit using the quasi-likelihood independence model information criterion (QIC).

Statistical analysis for the duration of mechanical ventilation outcome will be time (days) from study enrolment to the first ventilation free day (24 h free of mechanical ventilation support after extubation) as an outcome and will be performed using a competing risk approach. In this analysis, death will be considered as a competing event. Given delirium is operationalized as a dichotomy (either present or absent) over time, we will fit the models using general estimating equations (GEE). For binomial data, the most appropriate link function is the logit (logistic regression model) and, for incidence, the log (Poisson regression in log-linear model). Withdrawal of mechanical ventilatory support is not considered equivalent to death but the same as a medically planned extubation (i.e. patient who is determined to be “ready” for extubation by the medical care team). For those enrolled patients who undergo terminal withdrawal of ventilatory support, we will ascertain their vital status up to 30 days after enrolment in the study has ended.

For the above statistical analysis plan, we will report both unadjusted and false discovery rate (FDR) corrected *p*-values given FDR will account for multiple outcomes. Traditional approaches for controlling error rates in the presence of multiple comparisons include strong and weak control of familywise error rates, using techniques such as the Bonferroni correction [21]. The FDR approach has been shown to be more powerful than methods like the Bonferroni correction that control false-positive rates [22].

#### Secondary aims

The first secondary aim of the level of alertness (as assessed by the RASS) and sedative exposure between the two groups will be compared using two-sample *t*-test or Wilcoxon rank sum test as appropriate. Further analysis will be performed using repeated measures ANOVAs and multilevel models as in the case of the primary aim.

The second secondary aim is to compare VFDs in patients between the two groups defined as the mean/median number of days between successful weaning (usually extubation) from mechanical ventilation and day 28 after randomization. We do not count hours of days of non-invasive positive pressure support in the VFD calculation. We count VFD only during the current hospitalization. For patients receiving a tracheostomy to assist in weaning, “extubation” is defined as the first hour of a consecutive 24 h or more interval when the patient is on no positive pressure ventilator support. Patients can still be connected to the ventilator tubing to receive humidified gas or oxygen supplementation but not receiving PEEP or pressure support. Patients alive and on the ventilator at the time of hospital discharge (all of these would be ventilated via tracheostomy) are considered to be alive and on the ventilator for the remainder of the 28 days after randomization. Patients alive and off the ventilator at the time of hospital discharge are considered to be alive and off the vent for the remainder of the 28 days after randomization. Since one of the study sites (Mayo) has an internal chronic respiratory support unit, a patient transferred to that unit does not count as a hospital discharge and ventilator days are counted up to day 28. Some patients are “terminally extubated” but do not die immediately. In that case, we do not count the hours or days alive after terminal extubation as an “alive and vent-free day.” Some subjects have multiple episodes of intubation within the incident hospitalization in which case we aggregate all of those ventilator days in the VFD calculation. We do not include other ICU times of ventilation such as operating room (OR) or post-anaesthesia care unit. If an enrolled subject comes to the ICU from the OR intubated, then those ICU ventilator hours or days count towards VFD whether the procedure was emergency or elective as long as it occurred within the initial hospitalization. If the subject is discharged from the hospital and returns for more ventilator support, those days are not counted in the VFD calculation. Ventilator-free days will be compared using Wilcoxon rank sum test due to a possibility of non-normally distributed data.

### Interim analyses {21b}

No interim analysis is planned for this efficacy clinical trial.

### Methods for additional analyses (e.g. subgroup analyses) {20b}

There are two exploratory aims requiring analyses. The first exploratory aim is to compare post-ICU outcomes (physical/functional and psychological well-being; health-related quality of life) between patients randomized to self-management of sedative therapy and those receiving nurse-administered sedative therapy. The analysis will be performed using univariable and multivariable linear or logistic regression approach as appropriate.

The second exploratory aim is to compare immediate post-extubation recollections of ICU and to explore any relationships among cognitive experiences (CAM-ICU delirium presence/absence) and awareness (RASS scores) with mechanical ventilation complications (device disruption, self-extubation) and sedative exposure between the two groups. Statistical comparisons between the groups using total scores on the 25-item ICE questionnaire, as well as its 4 main categories, will be performed using two-sample *t*-test or Wilcoxon rank sum test as appropriate. In an event there are baseline imbalances between the two groups, we will use the linear regression approach to adjust for those variables with imbalances. The analysis will be performed separately for each of the three time points (24–48 h post-extubation, 3 months, 6 months).

We acknowledge that the study aims are relevant to both male and female patients and that the sexes may respond differently to the experimental intervention. Thus, we will analyse the data separately by sex allowing for the identification of sex-specific effects. The analysis will be performed using approaches as outlined above. Further, site-specific subgroup analysis will also be performed to account for potential differences in the management of mechanically ventilated patients. Power for such subgroup analysis will be limited due to the sample size.

### Methods in analysis to handle protocol non-adherence and any statistical methods to handle missing data {20c}

The analysis will be performed using an intention-to-treat approach. No imputations will be performed for missing data in this study.

### Plans to give access to the full protocol, participant-level data, and statistical code {31c}

Upon completion of all final analyses and acceptance of primary manuscripts for publication, a de-identified dataset will be made available upon request. Protocol information is available at www.clinicaltrials.gov.

### Oversight and monitoring

#### Composition of the coordinating centre and trial steering committee {5d}

There is no coordinating centre or trial steering committee for this clinical trial.

#### Composition of the data monitoring committee, its role, and reporting structure {21a}

An independent Data and Safety Monitoring Board (DSMB) oversees the data integrity, recruitment status, and safety of research subjects. The Midwest Area Research Consortium for Health (MARCH) DSMB is composed of two co-chairs, core members (11), ad hoc members inclusive of two physicians experienced in anaesthesiology and pharmacology per the sponsor’s request, and two non-voting staff members including a biostatistician. All members of the MARCH DSMB are independent from all members of the study investigators. The DSMB meets twice yearly with the study investigators and reviews all adverse events, serious adverse events, and accrual. A formal written report is formulated and then sent to the principal investigators detailing any necessary action or follow-up. For example, early in the conduct of this clinical trial, the DSMB requested the formulation of an independent adverse event adjudication committee from each participating research site. The members of the independent adjudication committee receive summary reports of any protocol defined adverse events/serious adverse event for their review and attribution of relatedness to the study protocol. Allocation of group assignment is not revealed to the adjudicators. These reports are subsequently submitted to the DSMB for their review.

#### Adverse event reporting and harms {22}

Adverse events (AEs) are detected in two ways. First, while the subject is in the ICU phase (i.e. 24 h following extubation, study withdrawal, transfer out of ICU or 24 h after completing 7 days on protocol), subject assessors review the EMR and talk with the bedside RN. Subject assessors can be either study RNs or study physicians. Their goal is to detect events in the prior 24 h such as hemodynamic changes, self-extubation, or new surgical procedures. Details are written on a case report form kept in the same binder as the subject assessment forms. Second, during EHR data abstraction for entry into the study database (which may occur weeks later), RN coordinators look for anticipated adverse events related to dexmedetomidine such as bradycardia and hypotension. Because the adverse events related to a sedation intervention are physiologically short-lived, we only record adverse events during the ICU phase, not for the entire time to the last follow-up at 6 months.

Critical care physician investigators at each site review the AE reports and complete a form indicating the severity by Common Terminology Criteria for Adverse Events (CTCAE) definitions. They also record “action taken”, “outcome”, “relationship to study”, and whether the event was a serious adverse event requiring expedited reporting to the overseeing agencies. After the first year of enrollment, the DSMB requested an additional level of adverse event interpretation. Each site chose two critical care physicians who were not part of the original study protocol or grant application. They are given the subject’s medical record number and date of the AE and asked to review the EHR (while blinded to the study assignment) for events around that date. They complete a form with their interpretation of whether the AE was mild, moderate, severe, life-threatening, or death and whether the relationship of the event to the study was not related, possible related, probable related, or definite not related. Those results are presented to the DSMB for their scheduled reviews. The institutional IRBs, NIH, and FDA do not require this independent review.

Events are reported to the respective Institutional Review Board, the National Heart, Lung and Blood Institute representatives, and the Data Safety and Monitoring Board per regulations and within the timeframes required based on the severity of the event and whether or not an event was unanticipated. Furthermore, a summary of all AEs/serious AEs is included in the annual continuing review reporting to the IRB at each participating site. The IRB then recommends the continuation of the study for another year and stipulates any further action needed, e.g., revision to the informed consent document if risk/benefit has changed.

#### Frequency and plans for auditing trial conduct {23}

An independent, external trial monitor audits the case report forms against the data entered into the REDCap® database, study eligibility, and informed consent documents. These audits are conducted on a twice-yearly basis, independent from the investigators and the sponsor.

#### Plans for communicating important protocol amendments to relevant parties (e.g. trial participants, ethical committees) {25}

Important protocol amendments will be communicated to potential trial subjects and/or their proxy representative in the informed consent document, most likely due to any new safety information about dexmedetomidine. We will also communicate any significant safety information to our local IRBs, the independent Data and Safety Monitoring Board, the National Institute of Heart, Lung and Blood Institute, and the Food and Drug Administration as indicated.

#### Dissemination plans {31a}

The results will be disseminated via published manuscripts and poster and podium conference presentations to healthcare professionals.

## Discussion

The conduct of this clinical trial has been severely hampered by the global COVID-19 pandemic. This includes but is not limited to the need to conserve personal protective equipment for direct patient care only. In both institutions, non-COVID-specific research activities were temporarily halted for several months (Mayo Clinic mid-March 2020 through early July 2020; University of Minnesota between mid-March 2020 and mid-September 2020). COVID-19 patients remained ineligible for the study until they were deemed non-contagious per hospital infection control regulations due to the protection of research staff from infection, re-deployment of nursing staff to support COVID-19 institutional efforts, and institutional priority on patient enrolment for completion of COVID-19-specific critical care clinical trials. In addition, elective surgeries were cancelled early in the pandemic while other surgeries may be postponed or reduced in the current environment due to patient hesitance to seek care or to enhance in-patient capacity. This has reduced the number of post-surgical patients who would have been routinely admitted to an ICU for respiratory management and who would have been eligible for study enrolment.

The practice of critical care and management of patients changes over the course of a 5-year clinical trial, in this case related to the management and use of sedatives as well as the use of ventilation techniques. For example, non-invasive respiratory support modalities have been used instead of invasive mechanical ventilation both at the onset (to avoid intubation) and during the resolution of respiratory failure to facilitate early extubation. This change in practice greatly limited the enrolment window as patients who otherwise fulfil the criteria (intubation, hemodynamic stability, ability to use the button for self-management) are often extubated within 12 h of meeting study criteria. It is imperative that research teams plan appropriately for how to manage practice changes that may impact the internal validity of a study protocol and the external validity of the research findings. As we learned from the COVID-19 pandemic, research teams should also have contingency plans for unforeseen circumstances that would impede the successful conduct of clinical trials.

### Trial status

Screening for this clinical trial began in December 2016 at the Mayo Clinic site and in April 2017 at the UMMC site. Currently, protocol version number 22 has been in effect since December 13, 2021. Enrolment of new research subjects is ongoing with anticipated completion of recruitment by October 30, 2022.

## Data Availability

LLC, CRW, MFT, DJS, OG, and JM will have access to the final trial dataset.

## Abbreviations

ICU: Intensive care unit
MN: Minnesota
EHR: Electronic health record
IRB: Institutional Review Board
PI: Principal investigator
CAM-ICU: Confusion Assessment Method-ICU
RASS: Richmond Agitation-Sedation Scale
FDA: Food and Drug Administration
ICE: Intensive Care Experience Questionnaire
KADL: Katz Activities of Daily Living
FAQ: Functional Activities Questionnaire
PHQ-9: Patient Health Questionnaire
PCL: Posttraumatic Stress Disorder Checklist Event Specific
SF-36: Short Form-36
REDCap®: Research Electronic Data Capture
APACHE: Acute Physiology, Age and Chronic Health Evaluation
CRFs: Case report forms
GEE: General estimating equations
OR: Operating room
VFD: Ventilator-free days
DSMB: Data and Safety Monitoring Board
MARCH: Midwest Area Research Consortium for Health
